# Targeting DCs for Tolerance Induction: Don’t Lose Sight of the Neutrophils

**DOI:** 10.3389/fimmu.2021.732992

**Published:** 2021-10-05

**Authors:** Florianne M. J. Hafkamp, Tom Groot Kormelink, Esther C. de Jong

**Affiliations:** Department of Experimental Immunology, Amsterdam University Medical Center, Amsterdam Institute for Infection & Immunity, University of Amsterdam, Amsterdam, Netherlands

**Keywords:** chronic inflammatory disorders, autoimmune disease, dendritic cell, neutrophil, tolerance, vitamin D3, corticosteroids, retinoic acid

## Abstract

Chronic inflammatory disorders (CID), such as autoimmune diseases, are characterized by overactivation of the immune system and loss of immune tolerance. T helper 17 (Th17) cells are strongly associated with the pathogenesis of multiple CID, including psoriasis, rheumatoid arthritis, and inflammatory bowel disease. In line with the increasingly recognized contribution of innate immune cells to the modulation of dendritic cell (DC) function and DC-driven adaptive immune responses, we recently showed that neutrophils are required for DC-driven Th17 cell differentiation from human naive T cells. Consequently, recruitment of neutrophils to inflamed tissues and lymph nodes likely creates a highly inflammatory loop through the induction of Th17 cells that should be intercepted to attenuate disease progression. Tolerogenic therapy *via* DCs, the central orchestrators of the adaptive immune response, is a promising strategy for the treatment of CID. Tolerogenic DCs could restore immune tolerance by driving the development of regulatory T cells (Tregs) in the periphery. In this review, we discuss the effects of the tolerogenic adjuvants vitamin D3 (VD3), corticosteroids (CS), and retinoic acid (RA) on both DCs and neutrophils and their potential interplay. We briefly summarize how neutrophils shape DC-driven T-cell development in general. We propose that, for optimization of tolerogenic DC therapy for the treatment of CID, both DCs for tolerance induction and the neutrophil inflammatory loop should be targeted while preserving the potential Treg-enhancing effects of neutrophils.

## Introduction

A distorted immune balance can culminate in various chronic inflammatory disorders (CID) such as allergic asthma and autoimmune diseases, e.g., rheumatoid arthritis, systemic lupus erythematosus (SLE), and type 1 diabetes (T1D). CID are generally characterized by loss of tolerance for either self-antigens or harmless environmental antigens, resulting in the continuous production of inflammatory mediators, such as interferon-γ by T helper 1 (Th1) cells or interleukin-17 (IL-17) by Th17 cells (1). In allergic asthma, a Th2 cell response dominates with associated cytokines IL-4, IL-5, and IL-13 (2). Generally, Th1 cells protect against intracellular pathogens like viruses and certain (myco)bacteria, whereas Th2 cells are indispensable for the eradication of helminthic pathogens (1, 2). Th17 cells are essential in the defense against fungi and bacteria, but they are pathogenic in the disease progression of multiple CID (1, 3, 4).

**Figure 1:**
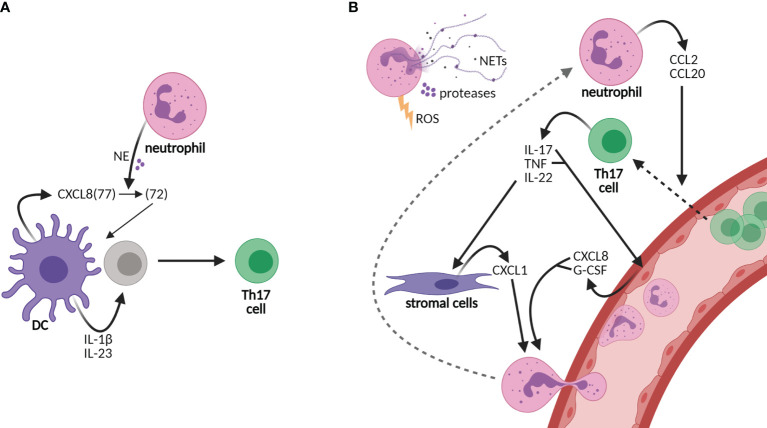
Neutrophils sustain an inflammatory loop of Th17 cell development and recruitment to tissues. **(A)** Neutrophils shape the adaptive immunity by influencing dendritic cell (DC)-driven T-cell development, e.g., Th17 cell development by cutting DC-derived CXCL8(72) into the short form CXCL8(72), which is required for Th17 cell development from human naive CD4 T cells. Other cytokines required for Th17 cell development are IL-1β and IL-23. **(B)** Neutrophils are recruited to tissues *via* granulocyte colony-stimulating factor (G-CSF) and the chemokines CXCL1 and CXCL8, among others, of which production is promoted by Th17 cells. CXCL8 and G-CSF are produced by epithelial cells upon stimulation by IL-17 and/or tumor necrosis factor (TNF), while CXCL1 is released from stromal cells upon IL-22 stimulation. In turn, neutrophils produce CCL2 and CCL20, ligands for receptors CCR2 and CCR6, respectively, on Th17 cells, thereby elevating the infiltration of Th17 cells in tissues. Furthermore, neutrophils contribute to tissue damage *via* their release of neutrophil extracellular traps (NETs), proteases, and reactive oxygen species (ROS). Neutrophils contribute to both the development of Th17 cells in lymph nodes and the perpetuation of inflammation in tissues *via* the recruitment of Th17 cells.

T-cell development is orchestrated by dendritic cells (DCs), specialized antigen-presenting cells, subsequent to the first-line response against pathogens by neutrophils, the major phagocytes of the innate immunity (5, 6). In recent years, compelling evidence has shifted our view of neutrophils from solely being short-lived first responders of the innate immune arm toward acting as accessory cells in adaptive immunity as well (6–8). Neutrophils promote the polarization of DC-driven T-cell development into Th17 cells *via* their granule content neutrophil elastase (NE) (9). DC-derived CXCL8 is processed into a short form by NE that promotes differentiation from human naive CD4 T cells to Th17 cells (9) (**Figure 1A**). In addition to their rapid recruitment to inflamed sites, neutrophils infiltrate draining lymph nodes *via* blood vessels, which was demonstrated in response to infectious agents, lysozyme immunization, or immune complexes in ovalbumin-immunized mice (10–15). At both sites, they are able to shape adaptive immunity by crosstalk with DCs and other immune or stromal cells, either by suppressing or by activating specific adaptive immune responses [reviewed in (6, 16, 17)].

In addition to IL-17, pro-inflammatory cytokine IL-22 and tumor necrosis factor alpha (TNF-α) are produced by Th17 cells. IL-22 induces the production of neutrophil-attracting chemokines by stromal cells, e.g., CXCL1 (18, 19). IL-17 and TNF induce the production of CXCL8 and granulocyte colony-stimulating factor (G-CSF) from epithelial cells, thereby increasing neutrophil activation and migration (20, 21) (**Figure 1B**). In turn, neutrophils chemoattract Th17 cells to the site of inflammation through the production of chemokines CCL2 and CCL20, ligands for the receptors CCR2 and CCR6, respectively, present on Th17 cells (22). Furthermore, neutrophils contribute to tissue damage and the overall inflammatory state in chronic diseases *via* the secretion of proteases and reactive oxygen species (ROS) and the formation of neutrophil extracellular traps (NETs) (23–25). NETs are composed of decondensed chromatin, histones, and granule proteins that serve as a useful tool to kill invading pathogens in host defense (6, 24). In rheumatoid arthritis and SLE, however, NET formation contributes to the disease activity as NETs are a source of autoantigens and they induce endothelial damage (26–28). Furthermore, NETs are released by neutrophils infiltrating the pancreas in T1D patients (25, 29). In the recent COVID-19 pandemic, NETs were also shown to contribute to disease severity (30, 31). Taken together, the recruitment of neutrophils to inflamed tissues and lymph nodes likely creates a highly inflammatory loop in CID through the induction of Th17 cells that should be intercepted to attenuate disease progression.

A counterbalance to inflammatory Th cell activity is provided by regulatory T cells (Tregs), which develop in the periphery from naive precursors upon antigen presentation in the presence of specific tolerogenic factors, such as transforming growth factor beta (TGF-β) and IL-10 (32). Tregs can inhibit Th cell function by cell–cell contact or the secretion of inhibitory cytokines. A defect in either the number or the function of Tregs has been demonstrated in various autoimmune disorders (32). Immune tolerance could be restored *via* the induction of tolerogenic DCs that drive Treg development in the periphery (33, 34). Immunomodulatory agents such as vitamin D3 (VD3), corticosteroids (CS), and retinoic acid (RA) show potency to induce tolerogenic DCs (35–38). A treatment approach to inducing tolerogenic DCs should also take the additional role of neutrophils in steering DC-mediated T-cell development into account. In this review, we discuss the effects of the tolerogenic adjuvants VD3, CS, and RA on both DCs and neutrophils and their potential interplay.

## DCs as Inducers of Peripheral Tolerance

Although it is evident that DCs are paramount in the orchestration of the immune response toward a tolerogenic state, a dogma emerged that functionally immature DCs are the tolerogenic DCs, whereas mature DCs are always immunogenic DCs that elicit responses against pathogens (39). A key feature of mature DCs is their ability to migrate to lymph nodes where they activate naive T cells by presenting antigenic materials. While migratory DCs transport pathogen-derived antigens, they may also carry self-antigens and induce a non-inflammatory response. Therefore, mature DCs can be divided into tolerogenic or immunogenic DCs that are clearly distinguishable by the expressions of different sets of molecules, as reviewed by Lutz et al. (39). Tolerogenic DCs should rather be characterized by specific markers found on tolerogenic DCs and the different expression levels of molecules in comparison to immunogenic DCs, as indicated below.

Generally, the expressions of major histocompatibility complex II (MHCII) molecules and the activation markers CD80 and CD86 are reduced in tolerogenic DCs compared to immunogenic DCs. Tolerogenic DCs have been shown to induce T-cell anergy *in vitro* (39, 40). However, in the presence of TGF-β, FoxP3^+^ Tregs are induced rather than anergic T cells (41, 42). Suppressed release of IL-12p40, a subunit of both IL-12 and IL-23, is required for the induction of Tregs, given that IL-12 alters the polarization of TGF-β cultured T cells from FoxP3^+^ Tregs toward Th1 cells (39, 43, 44). Another CD4^+^ Treg subset is that of Tr1 cells, characterized by a high expression of IL-10. The principal cytokine driving the generation of Tr1 cells is IL-10 (45). A specific human tolerogenic DC subset, termed DC-10, secretes high levels of IL-10, but no IL-12, and DC-10 potently induces Tr1 cells (46). TGF-β has no effect on Tr1 cell induction, while IFN-α, synergistically with IL-10, enhances Tr1 cell polarization (47). In addition to releasing TGF-β and IL-10, tolerogenic DCs express immunomodulatory molecules such as programmed death ligand 1 (PD-L1) and inducible co-stimulatory ligand (ICOSL), which induce Tr1 cells *via* their respective receptors, PD-1 and ICOS, on T cells (34, 39, 48–50). Another tolerogenic DC feature is the expression of the inhibitory receptor immunoglobulin-like transcript (ILT)-3, which has been associated with the increased generation of Tregs (46, 49, 51, 52). Furthermore, immunoregulatory enzymes can be upregulated by tolerogenic DCs, such as indoleamine-2,3-dioxygenase (IDO), which leads to a decreased T-cell proliferation and the induction of Tregs (53–55). Taken together, compared to immunogenic DCs, tolerogenic DCs are generally characterized by lower expressions of CD80/86, MHCII, and IL-12, while they secrete TGF-β and IL-10 and express tolerogenic markers such as PD-L1, ICOSL, ILT-3, and IDO.

## Neutrophils Shape DC-Driven T-Cell Development

**Figure 2:**
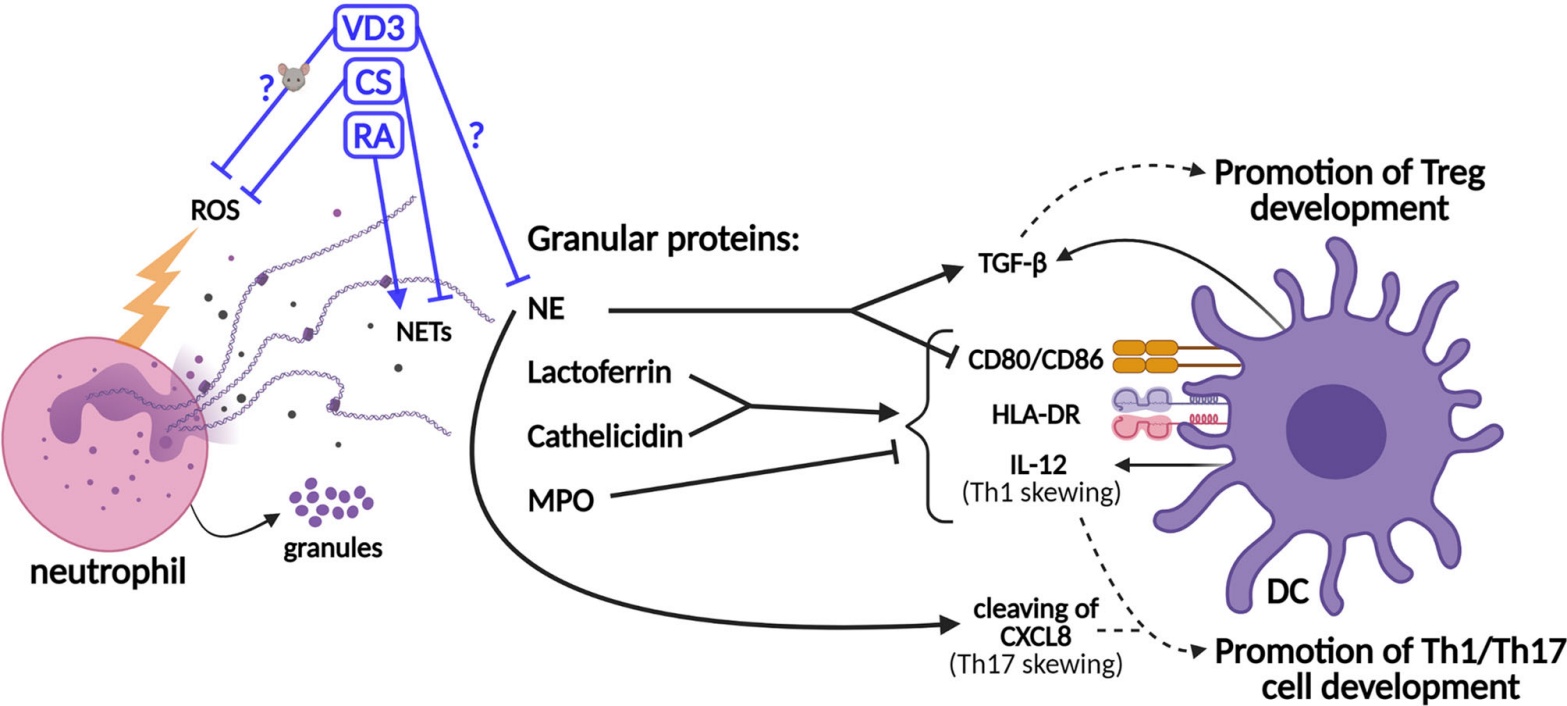
Schematic representation of the effects of neutrophil granule contents on dendritic cell (DC)-driven T-cell development and the effects of tolerogenic adjuvants on the functions of neutrophils. Granule proteins released by neutrophils modulate T-cell development *via* direct effects on DCs. The production of the co-stimulatory molecules CD80/86, the major histocompatibility complex II (MHCII) molecule HLA-DR (human leukocyte antigen—DR isotype), and IL-12 cytokine is upregulated by lactoferrin and cathelicidin, while myeloperoxidase (MPO) inhibits this. Neutrophil elastase (NE) cleaves CXCL8, which promotes Th17 cell development. However, NE also stimulates the secretion of TGF-β by DCs and inhibits the expressions of CD80/86. TGF-β promotes the development of Tregs, while IL-12 and cleaved CXCL8 promote the development of Th1 and Th17 cells, respectively. The effects of vitamin D3 (VD3), corticosteroids (CS), and retinoic acid (RA) on the release of neutrophil extracellular traps (NETs) (or NE specifically) and the generation of reactive oxygen species (ROS) are shown. The effects of VD3 and RA on (human) neutrophils are unclear and experimental data are scarce.

In addition to the modulation of T-cell responses by neutrophils *via* secreted mediators or cell–cell contact, neutrophils shape the adaptive immune response *via* the modulation of DCs (**Figure 2**) (7, 8, 16, 56). The half-life of neutrophils was shown a decade ago to be 5 days in human circulation (57). Previously, it had been described that the life span of neutrophil, originally estimated at 8 h in circulation, could be prolonged in inflamed tissues *via* activating signals such as microbial products or cytokines (58). When neutrophils become activated to release granule contents, such as lactoferrin, these proteins can affect DCs and, consequentially, T-cell polarization, as reviewed in Breedveld et al. and in Minns et al. (8, 16) Lactoferrin was shown to induce DC maturation of immature human DCs through upregulation of the expressions of CD83, CD80/86, and human leukocyte antigen (HLA)-DR isotype (59, 60). Consistently, the T-cell stimulatory capacity of DCs is increased by lactoferrin treatment (60). Furthermore, neutrophil-derived ROS may increase DC maturation, given that hydrogen peroxide increased the expressions of CD86 and HLA-DR on immature human monocyte-derived DCs (moDCs) (61). Moreover, hydrogen peroxide suppressed the Treg-inducing capacity of murine DCs (62). Therefore, the potential effects of neutrophil-derived ROS on DCs and T-cell development should be investigated.

In addition to lactoferrin, other granule components such as cathelicidin (LL-37), NE, and myeloperoxidase (MPO) modulate adaptive immune responses *via* their effects on DCs (**Figure 2**). Similar to lactoferrin, cathelicidin induces DC maturation and enhances the secretion of Th1-inducing cytokines (63). NE is required for the development of Th17 cells in humans, as DC-derived CXCL8(77) is cleaved into a short form that promotes Th17 cell polarization (**Figure 1A**) (9). On the other hand, MPO suppresses DC activation and IL-12 cytokine production (8, 16). Supporting the potential anti-inflammatory effects of neutrophils on DC-driven T-cell development, NE was shown to induce the production of TGF-β in human DCs *in vitro*, which favored polarization toward FoxP3^+^ Tregs (64, 65). Furthermore, NE impedes CD80/86 upregulation and the antigen-presenting ability of stimulated human moDCs (66). Hence, some granule contents were found to exert anti-inflammatory effects on DCs, thereby potentially even contributing to tolerance induction, while others stimulate DCs to facilitate Th1/Th17 cell development. Granule components decorate NETs to a different extent, as shown by Parackova et al. (67) The composition of NET differs substantially between pediatric T1D patients and healthy donors, with T1D NETs containing significantly more NE but less MPO and cathelicidin. T1D NETs induce significantly higher expressions of CD86 and HLA-DR on moDCs and elevate their production of the pro-inflammatory cytokines IL-6, CXCL8, and TNF when compared to healthy donor NETs (67). The relative abundance of granule proteins in NETs might alter the outcome of NET formation on adaptive T-cell responses, either promoting Th1/Th17 cell development or Treg development. Taken together, a delicate and intricate interplay between neutrophils (and their contents) and DCs orchestrates adaptive T-cell responses.

## Tolerogenic Adjuvants: Effects on DCs and Neutrophils

The use of 1,25-dihydroxyvitamin D3, calcitriol, the active form of VD3, is one of the most widely established protocols for the generation of tolerogenic DCs (36, 68). VD3 activates intracellular metabolic pathways in DCs *via* the PI3K/Akt/mTOR pathway that regulates glycolysis, retaining DCs in a more immature state with reduced expressions of CD80/86 and HLA-DR (36, 69, 70). VD3 reduces the production of IL-12 in DCs through suppression of NF-κB activity (70–72). Additionally, VD3 enhances the production of IL-10 by DCs and thereby favors the development of IL-10-producing Tregs (49, 69, 73). The expressions of the inhibitory receptors ILT-3 and PD-L1 on DCs are induced by VD3 (48, 49, 52, 70). Furthermore, we have previously shown that the migration of CD14^+^ dermal DCs, known for their tolerogenic effects, was increased by the intradermal application of VD3 in human skin explants (74). Dermal DCs primed with VD3 harbored less T-cell stimulatory capacity and altered T-cell polarization with increased Treg and reduced Th1 cell differentiation (74, 75).

Other well-recognized tolerogenic adjuvants are CS, which exert immunosuppressive effects *via* NFκB inhibition (36, 68, 76). Dexamethasone (Dex) is a commonly used synthetic CS. As shown for VD3, Dex reduces the expressions of CD80/86 on DCs and enhances their IL-10 production upon lipopolysaccharide (LPS) stimulation, while the release of IL-12 is suppressed. Correspondingly, Dex restrains the T-cell stimulatory capacity of DCs (48, 77). The tolerogenic DC features induced by Dex and VD3 largely overlap (36, 48) and may be complementary. Therefore, Dex and VD3 are also used simultaneously to induce tolerogenic DCs (35, 68, 78), given that both adjuvants endow DCs with a wide range of tolerogenic properties.

In addition to vitamin D, the active metabolite of vitamin A, namely, RA, is a known tolerogenic adjuvant. CD103^+^ DCs develop in response to RA, and these DCs promote tolerance to common harmless commensal bacteria in the gut (37, 79). RA-primed DCs induce the expression of the gut-homing receptor CCR9 on T cells, and they stimulate Tr1 cell development from naive T cells and FoxP3^+^ Treg development in the presence of TGF-β (37). Furthermore, RA decreases the expressions of CD80/86 and HLA-DR on human moDCs and induces the production of IL-10 in DCs (38). Although RA has been described to induce tolerogenic DCs (37, 79), substantial debate is ongoing on the potential pro-inflammatory role of RA. During infection or tissue damage, RA is capable of inducing a pro-inflammatory DC phenotype, characterized by the release of IL-12 and IL-23 (80). An increased IL-12 release is at odds with the preconditioned suppressed release of IL-12 for the induction of Tregs (43, 44). Therefore, caution is warranted when considering RA as an adjuvant for tolerance induction *in vivo* given that RA potentially has pro-inflammatory effects, dependent on the inflammatory environment.

While the effects of these adjuvants on DCs are widely described, studies on the effects of VD3 on neutrophils are scarce and largely contradictory. Neutrophils were shown to express mRNA of the vitamin D receptor (81). Handono et al. showed that VD3 treatment of neutrophils from SLE patients inhibited the externalization of NE during phorbol 12-myristate 13-acetate (PMA)-induced NETosis, but the study is limited, with only five patients and no healthy control comparison (82). On the other hand, VD3 was suggested to play a pro-inflammatory role in facilitating the neutrophil defense against certain viruses since VD3 induced NETs and the expressions of Toll-like receptor 7 and IFN-α (83). Additionally, elevated production of the neutrophil chemokine CXCL8 by human neutrophils was reported with 1-day pretreatment with VD3 prior to LPS stimulation, while the LPS-induced IL-6 and TNF release was unaffected by VD3 (84). However, this was contradicted by others (81). Moreover, *in vitro* VD3 priming of murine neutrophils reduced their immune complex-induced ROS release, while in human neutrophils, VD3 did not suppress PMA-induced ROS generation (81, 85). Taken together, additional studies are required to determine the effects of VD3 on various neutrophil functions. If future studies support the observation that VD3 reduces the release of NE (82), VD3 could hypothetically reduce NE-facilitated Th17 cell development, thereby intercepting the neutrophil inflammatory loop (**Figure 1B**).

The effects of CS on neutrophils have been extensively studied (86). CS were shown to prevent neutrophil apoptosis, which enables neutrophils to exert their functions for an extended period (87). A well-established anti-inflammatory effect of CS on neutrophils is their inhibitory effect on the release of CXCL8 (88–90), thereby decreasing neutrophil recruitment that could intercept the neutrophil inflammatory loop and tissue damage (**Figure 1B**). Furthermore, CS attenuate other neutrophil functions such as L-selectin-dependent migration, ROS production, and NET formation (91–93). Despite these *in vitro* effects of CS on neutrophils, resistance to corticosteroid treatment is an ongoing problem in the treatment of neutrophil-associated asthma and chronic obstructive pulmonary disease. The reduced expression of the glucocorticoid receptor (GR) in airway neutrophils and an elevated ratio of the inactive isoform GRβ *versus* the active GRα in neutrophils could underlie this resistance (88, 89, 94). Overall, given the anti-inflammatory effects of CS on neutrophils, CS such as Dex seems to be a suitable candidate as a tolerogenic adjuvant for the treatment of CID. Hypothetically, reduced NET and the concomitant release of NE could restrict the development of Th17 cells, and the well-established inhibitory effect of CS on CXCL8 is beneficial for restrained neutrophil recruitment to tissue. However, the potential Treg-promoting effects of NE *via* enhanced TGF-β production by DCs, as shown by Maffia et al. (64, 65), should not be neglected (**Figure 2**).

A pro-inflammatory effect of RA on neutrophils was demonstrated in the limited number of studies that have investigated RA on neutrophil function. One report showed that a short RA pretreatment of isolated human neutrophils inhibited *N*-formyl-methionyl-leucyl-fenylalanine (fMLF)-induced ROS production (95), while in another study, a 4-h pretreatment with RA prior to fMLF stimulation increased the production of intracellular ROS (96). Additionally, RA was found to increase the NET formation of these neutrophils (96). Furthermore, a study in rats demonstrated that the functions of neutrophils, including ROS generation and chemotaxis, were reduced in rats fed with a RA-deficient diet, which were restored when supplemented with vitamin A (97). In conclusion, although data on the effects of RA on neutrophils are scarce, no evidence for anti-inflammatory effects exist, and most reports actually demonstrated that RA is required for neutrophil differentiation and for optimal neutrophil function (98).

## Concluding Remarks and Future Perspectives

The induction of peripheral tolerance in autoimmune diseases or other CID should be antigen-specific, given that broad immunosuppression can give rise to recurrent infections, for which treatment is problematic (99). Current treatment approaches using tolerogenic DCs for autoimmune diseases are based on the *ex vivo* generation of tolerogenic DCs, named tolDCs, by re-education of patient-derived DC progenitors into antigen-specific tolDCs using immunomodulatory agents such as VD3 or Dex (35, 36). Clinical phase I and II trials using tolDCs have been conducted for T1D and multiple sclerosis, and phase I trials in Crohn’s disease and rheumatoid arthritis patients, as reviewed in Ten Brinke et al. (33) Due to the laborious and expensive nature of *ex vivo* tolDC generation, new approaches are in development for the *in vivo* induction of tolerogenic programs in DCs. These new *in vivo* approaches are focusing on selective targeting of disease-relevant autoantigens toward (inhibitory) DC receptors, resulting in an antigen-specific anti-inflammatory response (100). Alternatively, nanoparticles or liposomes can be targeted to DCs (76, 101). These carriers can be loaded with self-antigens and tolerogenic adjuvants, as discussed above (76, 101, 102). The addition of VD3 to a peptide-loaded liposome enhanced the development of Tregs in mice and decreased the differentiation of antigen-specific Th1 and Th17 memory cells (103). These data suggest that the development of both pathogenic Th1 and Th17 cells could be diminished by *in vivo* tolDC therapy, while the development of Tregs is enhanced, which could greatly ameliorate the disease course in patients suffering from various autoimmune diseases (1).

Even though the aim of tolerogenic therapy *via* DCs is to specifically target DCs with nanoparticles loaded with antigens and adjuvants, off-target effects could occur and the encapsulated adjuvant could influence the functions of other cell types. We discussed the effects of the commonly used tolerogenic adjuvants on neutrophils and the potential desired outcomes in view of intercepting the neutrophil inflammatory loop (**Figure 1**). Additionally, reduced neutrophil recruitment to tissue and the anti-inflammatory effects of these adjuvants could dampen neutrophil-induced tissue damage, e.g., by NET release, which is often associated with the exacerbation of CID (23, 24, 26–28). CS show the most profound anti-inflammatory effects on neutrophils, followed by VD3, but this requires further investigation. The possibility that neutrophils contribute to the development of Tregs, for example *via* the production of TGF-β, NE-induced TGF-β release by DCs, or *via* neutrophil-derived apoptotic bodies, should also be studied (64, 65, 104, 105). This would indicate that an intricate balance of dampening the inflammatory effects of neutrophils, such as NE release that facilitates the development of Th17 cells, while preserving their potential Treg-promoting effects may be desired for CID treatment. Alternatively, if such an intricate balance cannot be achieved and the overall function of neutrophils is dampened by treatment, the Treg-promoting effects could solely be provided by tolerogenic DCs. Analysis of the number and function of Tregs in patients with neutrophilic disorders, such as in chronic granulomatous disease (CGD) patients characterized by defective ROS production or in congenital neutropenia patients with mutations in the NE gene (*ELANE*) (9, 106), could provide valuable insights into the effects of neutrophils on Tregs. In CGD patients, the number and function of FoxP3^+^ Treg are not altered compared to that in healthy controls, while children with autoimmune neutropenia presented with a reduced frequency of FoxP3^+^ Tregs (106, 107). In conclusion, for the optimization and further development of tolerogenic DC therapy for the treatment of autoimmune diseases and other CID, neutrophils and their potential double-edged sword effects on DC-driven T-cell polarization should certainly be taken into account.

## Author Contributions

FH performed the literature search, wrote the manuscript, and created all figures with BioRender.com. TGK and EdJ critically read and carefully revised all versions of the manuscript, providing valuable guidance and insight. All authors contributed to the article and approved the submitted version.

## Funding

This work was supported by Amsterdam University Medical Center, University of Amsterdam.

## Conflict of Interest

The authors declare that the research was conducted in the absence of any commercial or financial relationships that could be construed as a potential conflict of interest.

## Publisher’s Note

All claims expressed in this article are solely those of the authors and do not necessarily represent those of their affiliated organizations, or those of the publisher, the editors and the reviewers. Any product that may be evaluated in this article, or claim that may be made by its manufacturer, is not guaranteed or endorsed by the publisher.
